# Modeling Pedestrian's Conformity Violation Behavior: A Complex Network Based Approach

**DOI:** 10.1155/2014/865750

**Published:** 2014-11-04

**Authors:** Zhuping Zhou, Qizhou Hu, Wei Wang

**Affiliations:** ^1^Department of Traffic Engineering, Nanjing University of Science and Technology, Nanjing 210094, China; ^2^School of Transportation, Southeast University, Nanjing 210096, China

## Abstract

Pedestrian injuries and fatalities present a problem all over the world. Pedestrian conformity violation behaviors, which lead to many pedestrian crashes, are common phenomena at the signalized intersections in China. The concepts and metrics of complex networks are applied to analyze the structural characteristics and evolution rules of pedestrian network about the conformity violation crossings. First, a network of pedestrians crossing the street is established, and the network's degree distributions are analyzed. Then, by using the basic idea of SI model, a spreading model of pedestrian illegal crossing behavior is proposed. Finally, through simulation analysis, pedestrian's illegal crossing behavior trends are obtained in different network structures and different spreading rates. Some conclusions are drawn: as the waiting time increases, more pedestrians will join in the violation crossing once a pedestrian crosses on red firstly. And pedestrian's conformity violation behavior will increase as the spreading rate increases.

## 1. Introduction

Traffic accidents involving pedestrians have become a major safety problem all over the world, particularly in developing countries. Several studies have examined gender and age differences in pedestrian behavior [1, 2]. Physical environment and personality traits characteristics are also likely to be related to differences in pedestrian crossing behaviors [3]. For example, self-identity, in this context characterized by individual's self-identification with being a safe pedestrian, has been shown to predict pedestrians road crossing intentions [4]. Another personality trait, conformity, involving characteristic willingness or a tendency to follow others' ideas, values, and behaviors, is also an important factor.

Conformity behavior, also known as herding behavior, refers to the individual's behavior tendency of following the group. This psychology phenomenon has been found prevalent in social life [5]. Most scholars thought the conformity psychology is an important reason for pedestrian's violation, but few of them studied the causes and mechanism of pedestrian's conformity behaviors. Through the questionnaire survey based on the theory of planned behavior, some deeper studies from Zhou Gonggang found that pedestrians would be much more likely to cross the road when some other pedestrians crossed than when all others waited for the “Green Man,” and pedestrians who perceived more behavioral control and had a greater tendency towards social conformity were more likely to exhibit positive intentions to cross on red [6, 7]. At present, the challenge for researchers is to investigate the process and causes for pedestrian's conformity in violation road crossing situation. An effective approach should be composed of basic theory and data analysis.

Complex networks have been successfully applied in many engineering fields. In this paper, complex network theory is used to analyze the structural characteristics and evolution rules of pedestrian network about the conformity violation crossings.

## 2. Literature Review

### 2.1. Network and Complex Network

In the 1960s, Erdos and Renyi proposed random graph theory to analyze the complexity of network topology. Random network is composed of *N* nodes and *P* × *N* × (*N* − 1)/2 edges, and *P* is the link probability between each pair of nodes. This classic mathematical theory can be seen as the foundation for complex network theory. The found of small-world and scale-free properties brings a new start for complex network study. When represented as graphs, some networks reveal a relatively small distance between each pair of nodes; that is, on average a small number of nodes separate them. This type of complex network, known as small-world, also shows high clustering; that is, if two nodes are connected to the same node, the probability that they are connected to each other is high [8]. Some other networks contain a few highly connected nodes. These networks are said to be scale-free [9]. Most real world networks in nature and social life have been shown to be scale-free.

Complex networks have attracted a great deal of attention in recent years. One of the main reasons why complex networks have become so popular is the flexibility and generality in representing virtually any natural structures, including those undergoing dynamic changes of topology [10]. Taking this into account, various studies have focused on how to describe a problem as a complex network, according to its topological characteristics and feature extraction.

Recently, the flow characteristics of the transportation system (such as traffic flows and pedestrian flow) become of primary interest in complex networks. In particular, traffic congestion and its dynamical relation to network structures have become a hot topic. But few studies combining the complex network and pedestrian behavior are presented in the reported literatures.

Inspired by the above research results, this paper would apply complex network theory to simulate pedestrian violation behavior.

### 2.2. Pedestrian Behavior Model

Pedestrian safety in urban areas is an issue of growing concern. Pedestrian behavior modeling is an important topic in the pedestrian safety research field. Researchers have built models to describe and simulate pedestrian movement characteristics since the 1970s. Previous methods for pedestrian behavior modeling can be classified into two main categories: microscopic and macroscopic models. In the last years, much more attention has focused on microscopic modeling, where each pedestrian is modeled as an agent. Microscopic models include social forces models, lattice gas (LG) model, cellular automata (CA) model, and artificial-intelligence-based models [11, 12]. The complex networks are used to represent self-organization phenomena, noise-induced ordering, and collective phenomena in different situations.

CA model uses a discrete space structure to simulate pedestrian walking behaviors including way change, step forward, and gap computation. In the model, each cell in the grid is represented by a state variable. A set of rules defines the cell's state according to the neighborhood of the cells, and a transition matrix is used to update the cell states in successive time steps. According to the rules, the lane which promotes forward movement best is chosen for sidestep movement. And the movement space of each pedestrian is based on the desired speed and the available gap ahead for forward moving. CA model is capable of effectively capturing collective behaviors of pedestrians who are autonomous at a microlevel [13, 14]. Similar to the CA model, each grid in the classical LG model has the same size, and each pedestrian just occupies a grid at each time step. Recently, LG model focuses on the interactions between pedestrians and vehicles. In addition, a social agent pedestrian model based on experiments with human subjects is a new research object [15].

## 3. Pedestrian Network Constructing

### 3.1. Modeling Approach

The core idea of complex network is to describe a system's macroscopic phenomena through exploring the microscopic individual's activities as well as the interactions between the individuals. Accordingly, complex network can be regarded as a bridge between microscopic individuals and macroscopic phenomena. In this paper, the theory of complex network is applied to capture pedestrian crossing behaviors at signalized intersections, especially when pedestrians are in a conformity situation.

Aims of this paper are to examine the pedestrian's conformity phenomena during the red signal time at intersections and to find out the spread rule of herding behaviors. The overall study process includes the following four steps: (1) use motion capture technology to collect the basic behavior data for constructing pedestrian network, (2) construct a pedestrian network and analyze the statistical parameters of the pedestrian network, (3) build a spread model of pedestrian's violation behavior using the approach of SI model, and (4) analyze the spread process of pedestrian's violation behavior based on the simulation results.

### 3.2. Network Model Constructing

Illegal pedestrians at signalized intersections can be well described by complex networks, where nodes represent the pedestrians, and links denote the relations or interactions among these pedestrians. According to their crossing behavior, illegal pedestrians can be divided into leaders and herding pedestrians. Leaders refer to the pedestrians who walk across the intersection firstly during the red light. Influenced by other illegal pedestrians, the pedestrians who commit violation accordingly are regarded as herding pedestrians. Based on the built pedestrian network, the mechanism of pedestrian dynamics when they are in conformity situation can be seen.

Pedestrian network has the following characteristics. (1) Directional: the relations or interactions among the pedestrians have direction, as pedestrians are only influenced by the front pedestrians and would not observe the behavior of pedestrians behind them in most cases. (2) Complex: nodes are complex, as pedestrian itself is a complex individual, whose behavior is influenced by personal factors, other pedestrian's behavior, and traffic environment. And the links are also complex, referring to the complexity, variability, and randomness of pedestrian behavior, as the relationship between the pedestrians is represented by their behaviors. (3) Increasing: under the impact of conformity psychology, pedestrian's violation behavior will increase constantly. The pedestrian network's nodes will grow dynamically.

### 3.3. Data Collection

Signalized intersections with large pedestrian volume and many illegal pedestrians are selected as the study sites in this paper. Pedestrian crossing behavior is studied through the data collected by a direct observation of pedestrian activities using two video cameras set up beside the crosswalks. Cameras are placed in relatively concealed locations, such as the nearby billboards and street trees, so that the presence of the camera would not affect the pedestrian's normal crossing behavior. In spite of pedestrian's crossing behavior such as violating or not, cameras also could film pedestrian's microscopic moving activities such as head movement, turning aside, looking, and saccade.

After the field study, researchers from Nanjing University of Science and Technology record the information obtained from the video. The detailed procedures to determine the behavior relationship between different pedestrians are presented as follows. Firstly, the pedestrian interaction region from the video is judged. It is shown in the literature [16] that observation range of the pedestrian is oval. And this result can roughly determine the pedestrians' interaction region. Then, observe the behavior of pedestrians who are in the interaction region to see whether there are some direct motion interactions between the pedestrians. If the pedestrian conducts some motions such as head movement, turning aside, looking, saccade, and talking, the pedestrian is seen to have interactions with other pedestrians. After that, researchers can record the useful data and take notes on the pedestrian's behavior and related information, such as signal cycle, signal time, pedestrian gender, and influencing pedestrian number.

The survey is carried out in the morning and evening peak hours. Video camera is placed at each crosswalk to record the expression and action of the pedestrian clearly. According to the basic method of the behavioral effects of relationship determination, the relationships between the pedestrians in different red light stage, different genders are recorded.  Table 1 shows the data record sheet for pedestrian interactions.

### 3.4. Network Characteristics of Pedestrian's Conformity Violation Behavior

In order to study the dynamics characteristics of the pedestrian's conformity violation behavior, the basic indicators of the networks need to be analyzed and calculated firstly. Given the proliferation mechanism and that the dissemination research goal is to explore the evolution of group behavior of pedestrians, pedestrians quantification of different types of individuals in the network status, and key individuals screened pedestrians group behavior, this paper intends to calculate the degree to analyze the topological characteristics of the conformity violation behavior.

**Figure 1:**
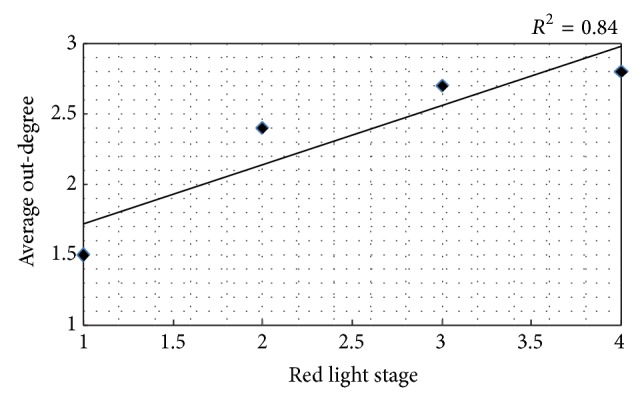
Relations between the average out-degree and the red light stage.

**Figure 2:**
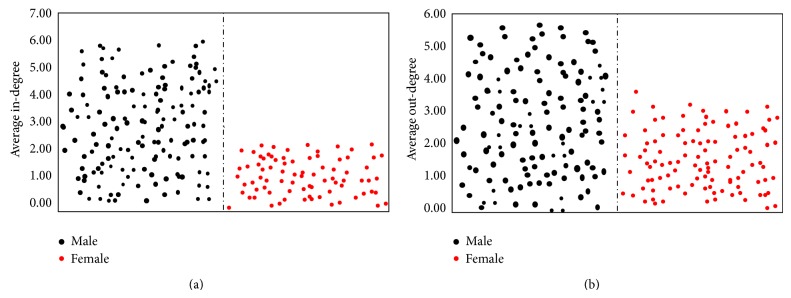
(a) Scatter diagram of average in-degree of female and male pedestrians in each signal cycle. (b) Scatter diagram of average out-degree of female and male pedestrians in each signal cycle.

Degree refers to the number of nodes connected to the other nodes. In the network, the degree of the node includes the out-degree and in-degree. Out-degree means the number of the nodes pointing to the others and in-degree means the number of other nodes pointing to node. And the average of all the nodes in the network is called the average degree of the network.

(*1) Average Out-Degrees in the Different Red Light Stage*. Through calculating the pedestrian average out-degree in 500 different signal cycles, the pedestrian average out-degree in stage one (0–10 s) is obtained. The average out-degree is 1.5, which means that the behavior of each pedestrian crossing street illegally could attract 1.5 other pedestrians following him. As the waiting time increases, the pedestrian average out-degree gradually increases. In stage four (50 s or more), the average out-degree of the pedestrian network is 2.8, indicating that pedestrians wait longer; the waiting pedestrians are more likely to commit violation when someone else does it firstly.  Figure 1 shows the correlation analysis results of the pedestrian average out-degree and the red light stage. *R* ^2^ is 0.84, indicating that the two variables are highly correlated. Therefore, in order to reduce the herd groups of illegal pedestrians, pedestrian signal should be set reasonable. For example, the time of red light should not be set too long.

(*2) Average Out-Degrees of Female and Male Pedestrians*. Through the average calculation of the out-degree and in-degree of male and female illegal pedestrians, the effect of gender factor on pedestrian's conformity behavior can be judged. It can be seen in Figures 2(a) and 2(b) that, in both in-degree and out-degree, the values of males are higher than females, which means that male pedestrians are more likely to follow others than females. This result is the same as the conclusion in literature [17]. The out-degree of males is also higher than female pedestrians, indicating that male's behavior not only is more likely to influence and attract other pedestrians, but also plays a key role in the illegal group than female pedestrians.

## 4. Spreading Model of Pedestrian's Illegal Crossing Behavior Based on Improved SI

### 4.1. SI Model

SI model is one of the classic models which are used to analyze the disease spread in biology. As this model can quantitatively analyze and numerically simulate the dynamics morphologically, the model is widely used in the complex networks field. In the SI model, each node is only in one of the two discrete states: one is healthy susceptible, named “Susceptible,” the other is infected which has infectiousness, named “Infective.”

Initially, the random selection of one or several of the network nodes is an infected node, and the others are healthy. At each time step, the nodes around the infected node could be infected with a certain probability. With the passing of time step, the evolution rules are parallelly conducted in the network. Computer viruses spreading on computer networks, rumors spreading in the community, and the diseases spreading in the population can be regarded as the behaviors spreading in the network. The process of pedestrian conformity behavior at signalized intersections is also consistent with the SI model. So SI model is used to analyze the pedestrian conformity behavior, to reveal the spreading characteristics, and to look for the effective control methods for reducing the conformity violation behavior.

During the red light time, pedestrians crossing the street could be divided into two categories by the movement characteristics: the pedestrians are walking (the illegal pedestrians) and the pedestrians are still waiting (in this paper, this pedestrian is defined as in a wait state). Once one of the crowded pedestrians crosses the street illegally, affected by the other's violation behavior, the pedestrians waiting to cross the street will think to choose crossing on red or not. These pedestrians are called in a “wait state.” Under the conformity mentality, part of the waiting pedestrians may follow the leader illegal pedestrian, while another part of the pedestrians follows the traffic laws and continues waiting until the pedestrian light turns green.

**Figure 3:**
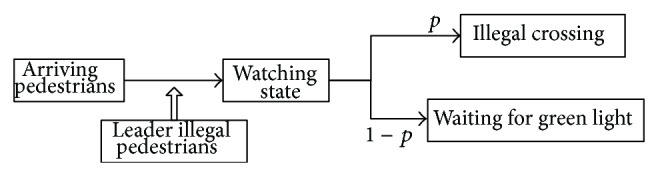
Framework of the conformity model.

Therefore, the pedestrians on crosswalk intersection could be divided into four categories by their behavior: the leader, the herding illegal pedestrians, the watching pedestrians, and the waiting pedestrians. The leader is the pedestrian who crosses on red firstly. Leader's illegal behavior begins to spread in the crowd. Pedestrians who receive illegal crossing street behavior information change into the watching state. Pedestrians in watching state may choose to commit violation or are still waiting for the green light following the impact forces such as traffic environment, psychological, social constraints. The detailed changing process is shown in Figure 3.

In this model, it is assumed that

1. pedestrians in a watching state have a certain probability *p* from watching state to crossing street illegal state and then have a certain probability 1 − *p* from watching state to waiting state;
2. for pedestrians crossing the street if they follow the leader, then the pedestrians' waiting state transforms to the crossing street illegal state;
3. at *t* time, the proportion number of pedestrians in crossing street illegal state and pedestrians in watching state is *i*(*t*) and *o*(*t*), respectively;
4. based on the principle of *k* neighbors, each illegal pedestrian can be observed by *k* pedestrians around him and impact the other pedestrians. *k* is a constant.

According to the assumption, each illegal pedestrian's behavior can be observed by *kl*(*t*) pedestrians around him. Because the number of pedestrians who are in the watching state is *No*(*t*), there are *p* *k* *No*(*t*)*i*(*t*) watching pedestrians who will cross the street illegally. As a result, the increasing rate of pedestrian crossing the street illegally is *p* *k* *No* *i*:

(1) $$\begin{array}{c} N\frac{di}{dt}=pkNoi. \end{array}$$

Also, as *i*(*t*) + *o*(*t*) = 1, at the initial time (*t* = 0), the proportion of illegal pedestrians is *i*_0_; then

(2) Ndidt=pkNi1−i,i0=i0.

Solve the equation, the results can be *i*_*t*_ = 1/(1 + (1/*i*_0_ − 1)*e* ^−*pkt*^).

### 4.2. Simulation Model of Pedestrian Violation Behavior

**Figure 4:**
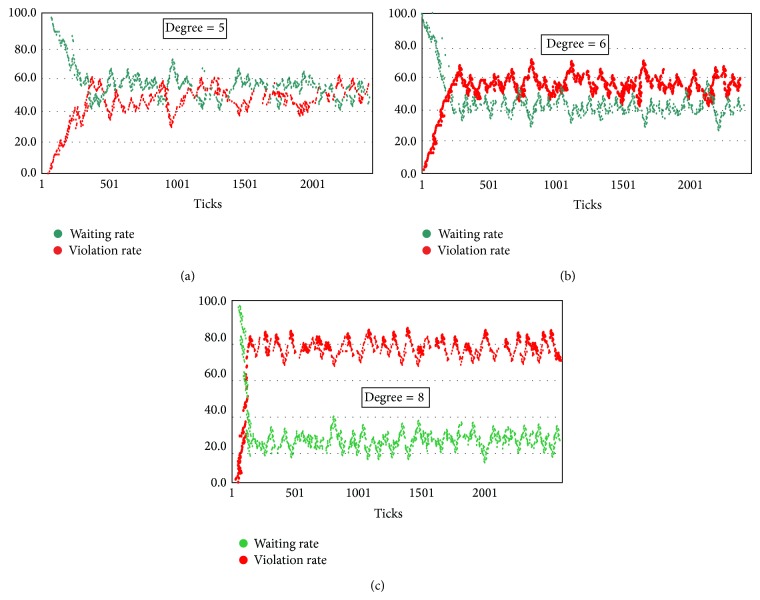
(a) Pedestrian violation behavior spreading trend in the degree of 5. (b) Pedestrian violation behavior spreading trend in the degree of 6. (c) Pedestrian violation behavior spreading trend in the degree of 8.

A complex system simulation software “NetLogo” is applied to simulate the spread model of pedestrian's violation crossing behavior.  Figure 4 shows the simulation results of the spread model. The red dots represent the pedestrians crossing the street illegally, while the green dots represent the pedestrians waiting for the green light. Through the simulation analysis, the spreading rules of violation behavior in different network structures are obtained. In addition, further analysis is proposed to study the factor of spreading rate in the pedestrian's crossing behavior in group.

The pedestrian violation behavior spread model based on improved SI is established. In the simulation process, to analyze the influencing factors of the behavior spreading, two key parameters are changed: the average degree of the network and the spreading rate. Spreading rate is set as 10%, and the spreading characteristics of violation behavior are simulated in the network when the average degree of the network is 2, 3, 5, 6, and 8. In addition, to analyze the factor of spreading rate, the spreading characteristics of pedestrian violation behavior are simulated when the average degree of the network is 6 and spreading rates are 10% and 15%.

#### 4.2.1. Spreading Characteristics of Violation Behavior in Different Network Structures

According to the simulation results, when the average degree of the network is less than 3, illegal behavior could not be spread on the pedestrian network. The leader's illegal behavior only can attract several pedestrians around him to cross the street illegally, but the illegal behavior could not be spread further. And other pedestrians crossing the street will not join in the illegal group. When the average degree of the network is close to or larger than 5, the crossing street illegal behavior can be spread in the network, which has produced the conformity effect.

In addition, detailed analysis shows that when the average degree of the network is 5, other pedestrians will gradually join into the ranks of crossing the street illegally when there is a leader illegal pedestrian. And about 50% of the pedestrians will cross the street illegally because of the conformity effect. When the average degree of the network is 6 or 8, the rate of pedestrians crossing the street illegally stabilized at 60% or 80%. It can be seen that (1) the spreading rule of illegal behavior has a steady effect, so the behavior spread of impact scope will eventually reach a steady state; (2) when the pedestrians' connectivity is much stronger (average degree is higher), the illegal behavior will spread more widely and fast.

#### 4.2.2. Spreading Characteristics of Violation Behavior in Different Spreading Rates

**Figure 5:**
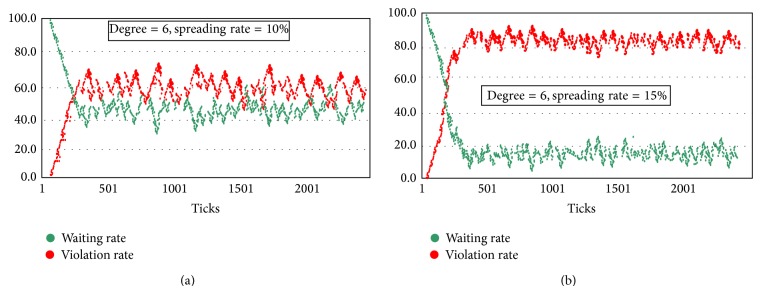
(a) Pedestrian violation behavior spreading trend (degree = 6 and spreading rate = 10%). (b) Pedestrian violation behavior spreading trend (degree = 6 and spreading rate = 15%).

Spreading rate is defined as the probability of the pedestrian whose illegal behavior is impacted by other pedestrians. Spreading rate can represent the amount of a person's conformity probability. Different simulation results when the average degree is 6 and the spreading rates are 10% (see Figure 5(a)) and 15% (see Figure 5(b)) are obtained. When the spreading rate is 10%, the rate of crossing street illegally eventually stabilizes at 60%, while when the spreading rate is 15%, the illegal crossing rates reach 80%. An important result is achieved: when the spreading rate is higher, the rate of pedestrians crossing street illegally is higher. The slight increase in spreading rates could cause a significant increase in pedestrian violation behavior spreading range. So improving pedestrian's safety awareness and compliance awareness can effectively reduce the probability of pedestrian violation behavior and improve pedestrian safety at signalized intersections.

## 5. Conclusions

Pedestrian conformity violation behaviors are common phenomena at the signalized intersections in China. Based on the theory of complex networks, the pedestrian's conformity violation behavior is studied in the paper. First, the network of pedestrians crossing the street is constructed and the degree distribution of the pedestrian network is analyzed. Then, using the basic ideas of SI model, pedestrians crossing street illegal behavior spread model is established. In addition, simulation method is applied to get the communication trends of pedestrian violation behavior in different network structures and different spreading rates.

It is concluded that as waiting time increases, more pedestrians will commit violation when the first pedestrian crosses the street illegally in the group. In addition, in both the in-degree and the out-degree, male pedestrians are higher than females. It means that male pedestrians are more likely to follow others than females, and male pedestrians' behavior is more likely to attract other pedestrians. Through analyzing the pedestrians illegal behavior spread rule, an important conclusion is found: as the spreading rate increases, the violation rate of pedestrians crossing street is higher. Therefore, improving pedestrians' safety awareness and compliance awareness can reduce the probability of pedestrian violations and improve pedestrian safety at signalized intersections.

## Figures and Tables

**Table 1:** Survey data record table.

Signal cycle	Red light stage	The number of the pedestrians	The pedestrians that have relationship with the former
	1—0–10 s; 2—10–30 s 3—30–50 s; 4—50 s	Female—F^*^; male—M^*^ ^*^is the number	Female—F^*^; male—M^*^ ^*^is the number
1	1	F1	F2, F3, M2, M5, and M6
1	2	F2	F1, F6, M2, M4, and M6
1	2	M1	F1, F5, M3, F4, and M8
⋮	⋮	⋮	⋮
